# Exploring the Role of Ubiquitin-Proteasome System in the Pathogenesis of Parkinson’s Disease

**DOI:** 10.3390/ph17060782

**Published:** 2024-06-14

**Authors:** Yiting Zhao, Man Lin, Fengguang Zhai, Jun Chen, Xiaofeng Jin

**Affiliations:** 1 Department of Chemoradiotherapy, The Affiliated People’s Hospital of Ningbo University, Ningbo 315040, China; 15267703251@163.com (Y.Z.); 17815917520@163.com (M.L.); 2 Department of Ultrasound Medicine, The Affiliated People’s Hospital of Ningbo University, Ningbo 315040, China; 3 Zhejiang Key Laboratory of Pathophysiology, Department of Biochemistry and Molecular Biology, Health Science Center of Ningbo University, Ningbo 315211, China; zhaifengguang21@163.com

**Keywords:** Parkinson’s disease, E3 ligase, deubiquitination enzyme, PD etiology, ubiquitin-proteasome system (UPS)

## Abstract

Parkinson’s disease (PD) is a prevalent neurodegenerative disorder among the elderly population. The pathogenesis of PD encompasses genetic alterations, environmental factors, and age-related neurodegenerative processes. Numerous studies have demonstrated that aberrant functioning of the ubiquitin–proteasome system (UPS) plays a crucial role in the initiation and progression of PD. Notably, E3 ubiquitin ligases serve as pivotal components determining substrate specificity within UPS and are intimately associated with the regulation of various proteins implicated in PD pathology. This review comprehensively summarizes the mechanisms by which E3 ubiquitin ligases and deubiquitinating enzymes modulate PD-associated proteins and signaling pathways, while exploring the intricate relationship between UPS dysfunctions and PD etiology. Furthermore, this article discusses recent research advancements regarding inhibitors targeting PD-related E3 ubiquitin ligases.

## 1. Introduction

**Figure 1:**
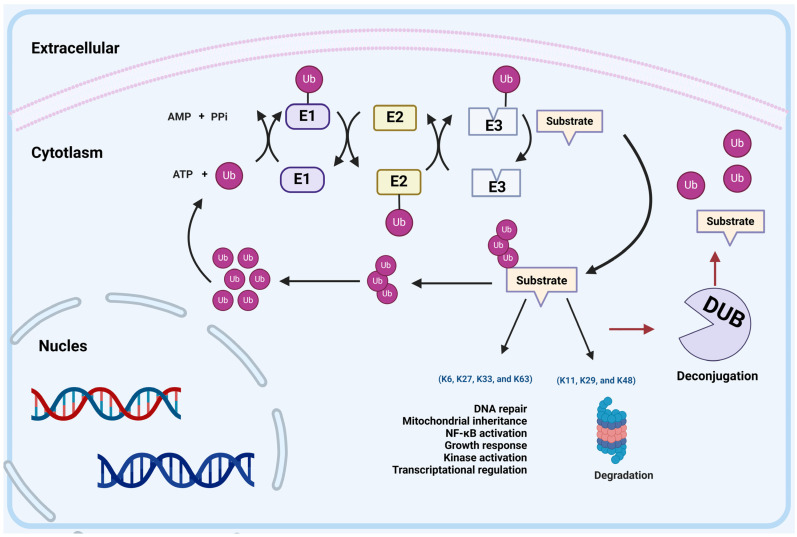
UPS-mediated protein degradation. The ubiquitin–proteasome system (UPS) consists of ubiquitin (Ub), E1 ubiquitin-activating enzymes (E1), E2 ubiquitin-conjugating enzymes (E2), E3 ubiquitin ligases (E3s), 26S proteasome and deubiquitinating enzymes (DUBs). E1 uses the energy of ATP hydrolysis to form a thioester bond between Cys in its active site and the C-terminus of ubiquitin, thereby activating Ub. The activated Ub is transferred from E1 to E2, forming a thioester bond. Subsequently, E3s bind nonspecifically to E2s and the target protein, thereby transferring Ub to the target protein. Different polyubiquitin linkage chains determine the fate of substrates: K48, K29, and K11 linkages direct proteins to the proteasome for degradation. K6, K27, K33, and K63 linkages regulate DNA repair, mitochondrial inheritance, NF-κB activation, growth response, kinase activation, or transcriptional regulation. In addition, ubiquitination is a reversible modification; the peptidase of DUBs can cleave Ub from the substrate protein.

Parkinson’s disease (PD) is a common neurodegenerative disease, ranking second in terms of incidence after Alzheimer’s disease. The onset of this condition primarily affects individuals aged 60 years and older. Patients with PD are mainly clinically manifested as tonic, resting tremor and bradykinesia and non-motor symptoms, such as sleep disturbance and cognitive decline. PD’s key pathological features are the presence of Lewy bodies (LBs) composed of α-synuclein (α-Syn) and the lack of dopaminergic neurons in the substantia nigra [1]. Most PD cases are sporadic and familial in only 5% to 15% of cases, with a well-defined chromosomal inheritance pattern. The onset of PD is related to factors such as age, environment, and genetic susceptibility [2]. Familial PD is affected by mutations in genes such as *Parkin* (*PRKN*), *glucocerebrosidase* (*GBA1*), *leucine-rich repeat kinase 2* (*LRRK2*), *vacuolar protein sorting-associated protein 35* (*VPS35*), *PTEN-induced putative kinase 1* (*PINK1*), and *F-box only protein 7* (*FBXO7*). Sporadic Parkinson’s disease is thought to result from the interaction of environmental factors and genetic predisposition. Notably, both sporadic and familial PD are associated with mitochondrial dysfunction. The ubiquitin–proteasome system (UPS) is a proteolytic system that controls protein degradation and plays a vital role in cellular protein homeostasis. UPS is intricately involved in the regulation of diverse biological processes, encompassing cell-cycle progression, proliferation control, cellular differentiation, molecular transport mechanisms, transcriptional regulation dynamics, signal transduction pathways, DNA damage repair mechanisms, immune responses, and inflammatory reactions. The UPS consists of ubiquitin (Ub), E1 ubiquitin-activating enzymes (E1s), E2 ubiquitin-conjugating enzymes (E2s), E3 ubiquitin ligases (E3s), 26S proteasome, and deubiquitinating enzymes (DUBs) [3]. Ubiquitination is a reversible post-translational modification that involves the conjugation of Ub to the substrate through a multi-step cascade mediated by E1, E2, and E3 enzymes. DUBs possess peptidase activity enabling them to cleave Ub from the substrate, process precursor forms of Ub, and modify Ub chains in order to counteract the actions of E3 enzymes. E1s uses the energy of ATP hydrolysis to form a thioester bond between Cys in its active site and the C-terminus of Ub, thereby activating Ub. The activated Ub is transferred from E1s to E2s, forming a thioester bond. Subsequently, E3s bind nonspecifically to E2s and the target protein, thereby transferring Ub to the target protein [4] (Figure 1). The full length of the Ub molecule contains seven lysine (Lys) sites (K6, K11, K27, k29, K33, K48, and K63), each of which can be ubiquitinated to form polyubiquitin linkage chains. Different polyubiquitin linkage chains mediate different signaling pathways to determine the fate of substrates [5]. Among them, K48 and K63 linkages are classical ubiquitin chain; the former mainly mediates the specific degradation signal of ubiquitinated proteins, and the latter mainly mediates the degradation signal of non-protein proteins [6]. K29 linkage is an important factor in controlling autophagy during cellular stress, and it can also alleviate the proteasomal stress response [7]. In addition, the ubiquitin chains of the K29/K48 and K48/K63 branches can also mediate efficient proteasomal degradation. The K29-selective ubiquitin binding domain reveals the structural basis of specificity and the heterotypic nature of k29 polyubiquitin [8]. K63 ubiquitylation triggers proteasomal degradation by seeding branched ubiquitin chains [9]. K11 linkage is not only a degradation signal but is also involved in the process of cell division and the regulation of transcription factor activity. It is worth noting that k11-linked ubiquitination can prevent and promote the induction of type I IFNs. K6 linkage can be involved in protein stabilization and other non-degradation processes, such as controlling DNA damage responses [10]. K27 linkage is closely related to the regulation of NF-κB and IRF pathways. Interestingly, it has been found that in zebrafish, both IRF3 and IRF7 can be decorated with k27-linked ubiquitination chains to promote their proteasome degradation. In addition, K27-linked ubiquitination also plays a role in antifungal signaling, where TRIM62 activates CARD9 through its K27-linked ubiquitination. K33 linkage plays an important role in protein-trafficking and negatively regulates innate immune responses (Figure 1) [7,11,12]. Notably, various studies have shown that the dysregulation of E3s leads to abnormal activation or inactivation of PD-related signaling pathways and the misfolding or abnormal accumulation of proteins, thereby affecting the occurrence and progression of PD. Recently, the aberrant function of numerous E3s and DUBs in PD have been found, some of which are actual PD inhibitory proteins, while others are PD promoters. Therefore, a comprehensive review focused on these PD-associated ubiquitination modification enzyme may provide a new direction for the future treatment of PD. This review introduces the research progress of PD-related E3s and related DUBs, elaborates upon their structure and function, and discusses the mechanisms of abnormal regulation of PD-related E3 ligase and DUB pathways. Emphasis is placed on the key role of E3s in the mitophagy and α-Syn-related signaling pathways. In addition, we integrate the current research situation and prospects for targeted therapies for abnormal E3s and DUBs in PD.

### 1.1. The Classification of E3 Ligases

To date, more than 600 E3s have been reported in humans, which are classified into the homologous to E6AP carboxyl terminus (HECT)-type E3 ligases, the RING-between-RING (RBR)-type E3 ligases, the really interesting new gene (RING)-type E3 ligases and UFD2 homology (U-box)-type E3 ligases, according to their structure and function [13,14]. E3s play an important role in PD, including regulating protein degradation processes, regulating mitochondrial function, and participating in the regulation of inflammatory responses. However, the pathogenesis of PD is very complex, and the specific mechanism of action of E3s still needs further research. Therefore, we summarized the E3s involved in the pathogenesis of PD and list them in Table 1.

#### 1.1.1. RING E3 Ligases

RING E3 ligases, the largest family of E3s, can be categorized into monomeric, homodimeric/heterodimeric, and multi-subunit forms [37]. Studies have shown that the activity of RING E3 ligases depends on their RING finger domain, which acts as a recruitment subunit for E2 enzymes [38,39]. Multi-subunit RING E3 ligases include Cullin-RING E3 ubiquitin ligases (CRLs) and the anaphase-promoting complex/cyclosome (APC/C) [40]. CRLs constitute the largest subgroup of RING E3 ligases and account for 20% of proteins degraded by the proteasome. They consist of substrate receptors, adaptors, RING finger proteins, and Cullin scaffolds [41]. The Cullin protein family mainly comprises Cullin1 (CUL1)-CUL7 but may also include Cul9 or p53-associated Parkin-like cytoplasmic protein (PARC) [42]. At the cellular level, CRLs actively regulate autophagy and protect nerve cells; however, their connection to neurodegenerative diseases remains unclear [43].

#### 1.1.2. U-Box E3 Ligases

The U-box-type E3 ligases, a subgroup of RING-type E3 ligases, can function as monomers or homodimers [44]. The C-terminal of the U-box-type E3 ligases contains a conserved U-box domain consisting of approximately 70 amino acids in yeast and humans. This domain interacts with Ub-conjugating E2 enzymes to directly transfer Ub molecules to target proteins [45]. CHIP functions as a U-box E3 ligase that interacts with several proteins associated with Parkinson’s disease (PD). Its structure comprises a tetratricopeptide repeat (TPR) domain and a U-box domain [46]. CHIP can function autonomously as an E3 ligase or form complexes with other E3s. It collaborates with chaperones to determine the chaperone-mediated degradation or refolding of unfolded proteins. Notably, as both an E3 ligase and co-chaperone, CHIP is responsible for ubiquitinating and degrading the proteins crucial in neurodegenerative diseases [47]. It is involved in the aggregation and degradation of α-Syn, the primary component of the Lewy bodies (LBs) associated with PD [48]. Additionally, Parkin and PINK1 are also linked to PD. CHIP interacts with α-Syn to enhance its degradation and positively regulate Parkin’s E3 activity [21]. Also, UFD-2 is an important U-box E3 ligase. UNC-45 acts as a co-chaperone along with the general chaperones, Hsp70 and Hsp90, in the process of promoting the folding of the motor domain of myosin molecules. The UNC-45 chaperone can act as an adaptor protein of UFD-2 to poly-ubiquitinate unfolded myosin, which may play a role in maintaining muscle-cell protein homeostasis [49]. Previous work has shown that a fusion protein bearing a “nonremovable” N-terminal Ub moiety is short-lived in vivo, with the fusion’s Ub functioning as a degradation signal. The proteolytic system involved, termed the UFD pathway (Ub fusion degradation), and UFD-2, are involved in this pathway [50]. While the association between UFD and PD may not be definitive, some studies suggest that the abnormal aggregation of proteins and disruption of degradation pathways may be associated with the onset and progression of neurodegenerative diseases. Therefore, the specific molecular mechanism of UFD and the development of Parkinson’s disease still need to be further explored and studied.

#### 1.1.3. HECT E3 Ligases

The human proteome comprises 28 distinct types of HECT-type E3 ligases, which can be classified into three groups based on the architecture of their N-terminal domains, as follows: the Nedd4 family, the HERC family, and other HECTs [51]. These E3 ligases possess a C-terminus HECT domain consisting of N and C lobes with a molecular weight around 40 kDa. The C lobe forms an intermediate sulfur–lipid bond with Ub through its active cysteine site to directly label protein targets for ubiquitination. On the other hand, the N lobe specifically recognizes ligase substrates and interacts with E2 enzymes [52]. Recently, increasing evidence supports the crucial role of HECT E3 ligase in various human pathologies, including neurodegenerative diseases and cancer [53]. Given that oxidative stress is a triggering factor for PD, it is plausible that the HECT E3 ligase HACE1, involved in oxidative stress, may directly or indirectly contribute to the development of PD. In Zang et al.’s study, knockdown of HACE1 led to microglial activation and accelerated neuronal death both in vitro and in vivo. Additionally, the expression level of HACE1 decreased with age in the brains of A53T transgenic mice. These findings confirm the neuroprotective effect of HACE1 in PD [54].

#### 1.1.4. RBR E3 Ligases

The RBR E3 ligase is an emerging E3 ligase isoform that has identified 14 RBR E3s in the human genome [55]. Unlike other types of E3 ligases, the RBR E3 ligase consists of the central intermediate ring (IBR), a catalytic cysteine, and RING1 and RING2 fingers. Specifically, the interaction between Ub-loaded E2 enzymes and RING1 promotes Ub transfer to cysteine on RING2, forming thioester intermediates which then transfer Ub to target proteins [56]. In terms of the Ub transfer mechanism, RBR E3 ligases exhibit the typical characteristics of both HECT and RING E3s. Additionally, Parkin is a well-known member of the RER E3 ligases. Its mutation is the most common cause of autosomal recessive PD and will be discussed in more detail later in this article [57].

### 1.2. The Classification of Deubiquitinating Enzymes (DUBs)

More than a hundred DUBs have been reported, consisting of seven subtypes: Ub-specific proteases (USPs), Ub C-terminal hydrolases (UCHs), ovarian tumor proteases (OTUs), Machado-Joseph disease proteases (MJDs), zinc finger-containing Ub peptidase 1 (ZUP1), motif interacting with Ub-containing DUB family (MINDY) and JAB1/MPN/Mov34 metalloenzymes (JAMM) [58]. USPs, UCHs, OTUs, MJDs, ZUP1 and MINDY are cysteine peptidases while JAMM utilizes zinc metalloproteinase domains to disrupt ubiquitin-substrate bonds [59]. Additionally, Table 2 provides a comprehensive summary of PD-associated DUBs.

The USPs, characterized by their USP domain, consisting of finger, thumb, and palm sub-domains, are the largest family of DUBs. The catalytic site is located between the thumb and palm domains, while the finger domain interacts with the distal Ub [74]. As a cysteine protease, the USP’s catalytic capacity primarily relies on cysteine’s nucleophilic attack at the catalytic site. USPs can impact PD by regulating proteasome activity and autophagy levels as well as by controlling Parkin’s stability and activity [75]. Bingol et al. demonstrated that while USP30 and USP15 counteract Parkin, Parkin loss of function drives PD progression. Therefore, the activities of USP30 and USP15 in antagonizing Parkin would be expected to promote PD phenotypes [62]. USP14 affects the progression of PD by regulating mitophagy [61]. Additionally, USP members like USP8 have close relationships with PD. In summary, most USPs involved in PD mediate its development by altering mitophagy.

The UCH deubiquitinases subfamily consists of four members, as follows: BAP1, UCH-L1, UCH-L3, and UCH-L5. Among them, UCH-L1 is a crucial neuronal deubiquitinase that hydrolyzes the C-terminal ester and amide of Ub to degrade multiple Ub linkage chains back to Ub monomers [76]. It is specifically expressed in neurons and plays a critical role in Parkinson’s disease (PD) by stabilizing key pathological proteins. Damage to UCH-L1 can impair proteasomal function, leading to the increased accumulation of damaged or misfolded proteins and ultimately resulting in neuronal cell death. Moreover, UCH-L1 interacts with and serves as a substrate for Parkin, an E3 ubiquitin ligase associated with PD [76].

The OTU subfamily in humans consists of 16 members and is involved in various human diseases, including cancer, inflammation, neurodegeneration, and viral infections [59,77]. Abnormal expression and genetic alterations of DUBs play a role in diseases such as PD [78]. For example, OTUD3 plays a crucial role in iron deposition in the substantia nigra of PD, while OTUB2 may have potential implications for PKM2 activity and neuroprotection in PD [71,73].

Ataxin-3, a member of Parkin, interacts with mutations of *Ataxin-3* (*ATXN7L3*)*,* promoting the autophagy degradation of Parkin. Patients with Machado–Joseph Disease may exhibit features of PD. BRCC3, a member of JAMMs, plays a role in neuronal inflammation through promoting NLRP3 inflammasome in PD [64].

## 2. Roles of E3s in PD

### 2.1. Relationship between Ubiquitination and Autophagy in PD

Autophagy is a cellular process in which a cell sequesters and encapsulates its own proteins or organelles within vesicles, which subsequently merge with lysosomes to form autophagic lysosomes. These lysosomes degrade the enclosed contents, playing a pivotal role in cellular metabolism and organelle renewal. Autophagy can be classified into the following three types, based on the manner in which degraded substances are sequestered: macroautophagy, microautophagy, and chaperone-mediated autophagy (CMA) [79]. Currently, it is widely believed that PD is linked to oxidative stress, and mitochondria play a crucial role in cellular response to oxidative stress. Mitochondria are dynamic organelles with their own genome and protein synthesis mechanisms, constantly undergoing fission and fusion within cells to regulate their number and morphology [80]. Mitophagy, a subtype of macroautophagy, is the most extensively studied form of organellar autophagy. It plays a crucial role in regulating mitochondrial quantity and maintaining energy metabolism by eliminating dysfunctional or redundant mitochondria [81]. During mitochondrial metabolism, accumulated ROS alters the permeability of the mitochondrial membrane, leading to cytochrome c release and ultimately triggering cell apoptosis. In a physiological state, cells selectively degrade damaged mitochondria through mitophagy to maintain homeostasis. Impaired mitophagy results in the accumulation of aging mitochondria within cells, causing cell degeneration and even death. Importantly, abnormal mitophagy function alone can cause PD by driving neuronal dysfunction [82,83,84]. Recently, the relationship between ubiquitination and mitophagy has gained increasing attention. E3 ligases like Parkin, MARCHF5, and MUL1 have been found to regulate ubiquitination and mitophagy in driving PD [85].

#### 2.1.1. Parkin

##### Parkin and Mitophagy

**Figure 2:**
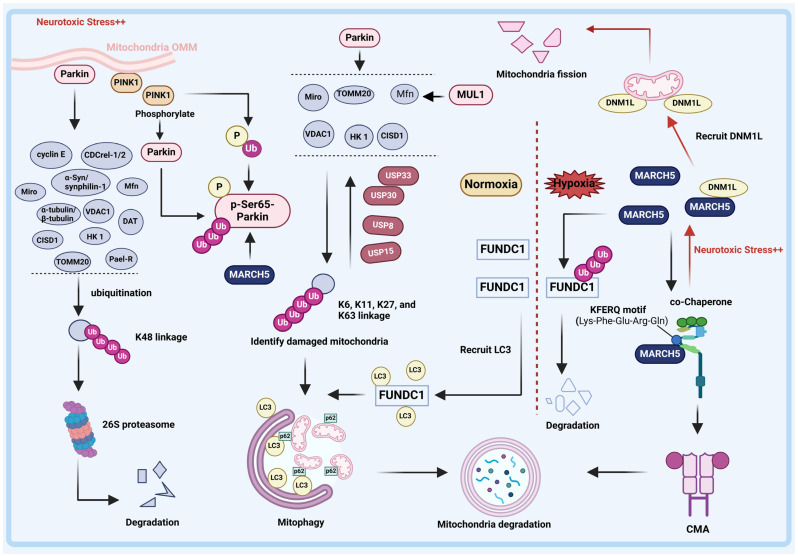
Relationship between E3 ligases and DUBs with mitophagy in PD. PINK1/Parkin pathway. Under neurotoxic stress, PINK1 accumulates in the outer mitochondrial membrane (OMM) and then recruits Parkin. Parkin-mediated modification of the K48-linked ubiquitin (Ub) linkage chain degrades the substrates by the proteasome, which partially inhibits the development of PD. Additionally, following MARCH5 ubiquitination-mediated modification of Parkin, PINK1 phosphorylates Parkin and Ub at Ser65, thereby activating Parkin on the OMM. Subsequently, the OMM proteins, including VDAC1, Mfn, Miro, HK 1, CISD1, and TOMM20 on the OMM are ubiquitinated by Parkin to form Ub linkage chains connected mainly via K6, K11, K27 and K63 linkages. Ubiquitinated OMM proteins isolate damaged mitochondria, allowing them to enter mitophagy programs. Significantly, USP30, USP15, USP33, and USP8 are capable of abrogating Parkin-mediated ubiquitination of OMM proteins. Then, mitophagy-associated adapter protein p62 accumulates in the OMM and recognizes the Ub chain, inducing the ubiquitinated products binding to LC3, ultimately initiating the mitophagy. In addition to being regulated by the PINK1/Parkin pathway, the OMM protein Mfn was also determined to be directly ubiquitinate by E3 ligase MUL1 to affect mitophagy. MARCH5 pathway. E3 ligase MARCH5 is a chaperone-mediated autophagy (CMA) substrate. Under normal conditions, MARCH5 is recognized and degraded by CMA. Neurotoxic-stress-induced accumulation of MARCH5 promotes the recruitment of dynekin-like (DRP1) into mitochondria, triggering the excessive fission of mitochondria in dopaminergic neurons. In addition, MARCH5 is a protective factor under stress. Under hypoxia induction, the OMM protein FUN14 domain containing 1 (FUNDC1) is activated in mammalian cells and facilitates mitochondrial autophagy by interacting with LC3.

The Parkin protein is a member of the RBR E3 Ub proteins, characterized by a ligase Ub-like structure (Ubl) at the N-terminus, three novel genes (RING0, RING1 and RING2), an intermediate RING structure (IBR), and a repressor structure (REP) at the C-terminus. Parkin’s C-terminus allows self-ubiquitination and collaboration with Ub-coupled enzymes UbcH8, Ubc6, Ubc7, and UbcH7 for substrate degradation [86]. Initially identified as a genetic locus for autosomal recessive Parkinson’s disease (ARPD), various deletions and point mutations in *Parkin* (*PRKN*) have been found in ARPD patients [87]. Notably, Parkin possesses a dual-acting E3 ligase that modifies the Ub chain linked to k48 and k63. The k48-linked Ub chain promotes substrate degradation by the proteasome, while the k63-linked Ub chain disrupts proteasome function, promoting substrate stability and aggregation, thereby participating in LB-like inclusion formation [88]. Currently, Parkin has been found to target various proteins as substrates, including synphilin-1 (an α-Syn interacting protein) and cyclin E (a cell-cycle protein) (Table 3) (Figure 2) [89]. The absence of Parkin can lead to the accumulation of these substrates, resulting in neuronal dysfunction, and, ultimately, death [90].

In addition to its role as an E3 ligase, Parkin also plays a crucial role in mitophagy (Figure 2). Under normal conditions, PINK1 protects brain neurons by phosphorylating neuroproteins. When stressed, PINK1 accumulates in the outer mitochondrial membrane (OMM) via outer membrane translocase (TOM), and then phosphorylates Parkin at Ser65, inducing a conformational change that activates and recruits Parkin to the OMM [96,97]. Furthermore, prior to phosphorylating Parkin, PINK1 phosphorylated Ub with a conserved phosphorylation site shared by Parkin. PINK1-mediated phosphorylation of Parkin and Ub is a crucial signal to trigger mitophagy [97]. Subsequently, the OMM proteins, including voltage-dependent anion channel 1 (VDAC1), mitofusion (Mfn), mitochondrial Rho (Miro), Hexokinase I (HK 1), CDGSH iron sulfur domain 1 (CISD1), and translocase of outer mitochondrial membrane 20 (TOMM20) on the OMM are ubiquitinylated by Parkin to form Ub linkage chains connected mainly via K6, K11, K27 and K63 linkages [94,95]. These OMM proteins isolate damaged mitochondria from a healthy mitochondrial network, allowing them to enter subsequent mitophagy programs. Then, the mitophagy-associated adapter protein, p62, accumulates in the OMM and recognizes the phosphorylated Ub linkage chain, inducing the ubiquitinated products binding to MAP1LC3B/LC3B (LC3, a mitophagy marker protein) to be recruited into autophagosomes, thus initiating the formation of autophagolysosomes to degrade mitochondria [98,99]. In addition, FBXO7, a fragment of Skp-Cullin-F-box (SCF) E3 ubiquitin ligase, has been reported to be involved in mitochondrial autophagy as a helper protein of Parkin. Fbxo7 promotes Parkin translocation to depolar mitochondria, a process that requires the involvement of PINK1 [100]. Therefore, a lack of Parkin or PINK1 may lead to the accumulation of dysfunctional mitochondria, which can increase oxidative stress.

Until now, mutations in *Parkin (PRKN)* are known to be closely related to the onset of PD [89]. More than 170 mutations have been identified in *PRKN* and are pathogenic events in the progression of PD [101]. *PRKN* mutations include point mutations and deletions, and duplications ranging in size from a few base pairs to multiple complete exons, with exon 2-, 7-, and 9-point mutations being multiple, and exon 3 and 4 deletions being the most common. Studies have demonstrated a certain correlation between the mutation type of *Parkin* (*PRKN*) and the genetic subtype of PD, with familial PD predominantly characterized by large exon fragment deletion mutations [102]. In contrast, sporadic PD is mainly based on point mutation of the *PRKN* [103]. Notably, Parkin’s mutation at the Ser65 site has received widespread attention due to the importance of phosphorylation at Ser65 for its activation in vivo. The mutation of *PRKN* at the Ser65 site results in functional loss, weakening mitophagy, and an increase in PD-associated substrates. Specifically, the *PRKN* Ser65 mutant evades phosphorylation by PINK1, thereby impairing its ability to interact with mitophagy-associated proteins, including VDAC1, Mfn, Miro, HK 1, CISD1, and TOMM20 [104]. However, Kane et al. suggested that there is another cytosolic substrate, Ub, which is involved in Parkin activation. Although *PRKN* mutation at the Ser65 site prevents phosphorylation by PINK1, the Parkin S65A mutant still translocated to mitochondria in a PINK1-dependent process. This was attributed to Ub contributing to the activation of Parkin E3 ubiquitin ligase activity, which partly explains why Parkin Ser65 mutant still participates in mitophagy [97].

The Parkin S65A mutant can induce PD by promoting mitochondrial accumulation, which may be partially reversed by Ubiquitin (Ub). However, the comprehensive mechanism underlying PD caused by the Parkin Ser65 mutant requires further investigation. The involvement of identified substrates in the pathogenesis of PD resulting from Parkin mutations or deletion remains unclear. Notably, animal models lacking Parkin do not exhibit substrate accumulation, suggesting a potential regulatory role of Parkin on its substrates. Therefore, additional studies are needed to establish conditional Parkin knockout in model mice to confirm this regulatory effect [105,106].

Although the current research on *PRKN* mutation-induced PD pathogenesis is limited, it remains crucial to conduct a thorough analysis in the future due to the high mutation rate of *PRKN* in ARPD. Moreover, while *PRKN* mutations can only partially explain PD pathogenesis, it is imperative to comprehensively screen for PD-related genes in diverse populations and explore other prevalent pathogenic genes.

#### 2.1.2. SIAH

##### SIAH and Mitophagy

The seven in absentia homolog (SIAH) family, consisting of SIAH1, SIAH2, and SIAH3, is a group of RING E3 ligases [107]. The sequences of SIAH1 and SIAH2 are 86% identical and share high structural homology. SIAH family proteins are composed of an N-terminal catalytic RING finger domain implicated in binding to E2s, two zinc finger domains, and a C-terminal substrate-binding domain (SBD) domain that binds to substrates. Notably, due to the lack of a cyclic domain, SIAH3 is an inactive E3 ligase [108]. Recent studies have shown that SIAH family proteins participate in the regulation of mitophagy and play a role in the pathogenesis of PD by degrading PINK1. In PD brains, SIAH3 colocalizes with PINK1 and initiates the inactivation of PINK1, causing a cascade of oxidative stress and apoptosis inside mitochondria. In addition, another member of the SIAH family, SIAH1, targeting the ubiquitination of PINK1, was found to promote the degradation of PINK1 by the proteasome, which hinders the regular renewal of damaged mitochondria [16]. Overall, the interaction between SIAH1 and SIAH3 with PINK1 collectively contributes to the onset of PD, suggesting that inhibiting their activity could potentially delay PD progression. However, caution should be exercised as loss of function in SIAH1 has been associated with Buratti–Harel syndrome [109].

#### 2.1.3. MUL1

##### MUL1 and Mitophagy

Mitochondrial E3 Ubiquitin Protein Ligase 1 (MUL1), a mitochondrial outer membrane protein, exerts regulatory control over mitochondrial fission. The MUL1 protein is classified as a RING E3 ubiquitin ligase with two transmembrane domains at the N-terminus and features a RNF structure at the C-terminus [110]. The transmembrane (TM) domains enable MUL1 to anchor to the mitochondrial OMM and enable it to participate in mitochondrial fission. Previous studies have found that MUL1 is associated with multiple physiological processes, including the regulation of mitochondrial fusion, activation of NF-kB pathways, innate immunity and antiviral signaling, and protease-dependent apoptosis [111,112,113].

In PD, MUL1 was identified as a promotion factor via restrained mitophagy [17]. As is widely acknowledged, the preservation of mitochondrial integrity is delicately regulated by two opposing mechanisms: mitochondrial fusion and fission. Mfn1 and Mfn2 predominantly facilitate mitochondrial fusion, whereas Dynamin-associated protein 1 (DRP1) primarily governs mitochondrial fission [110]. Notably, elevated Mfn2 levels impair endoplasmic reticulum (ER)-mitochondrial contacts and promote mitophagy. The direct ubiquitination of Mfn by MUL1 has been experimentally demonstrated. Specifically, the deletion or mutation of *MUL1* results in impaired mitophagy, whereas maintaining the activity of MUL1 enhances mitophagy [17,18].

In addition to inhibiting mitophagy by degrading Mfn, MUL1 also participates in mitophagy by stabilizing PINK1. In the PINK1/Parkin-mediated mitophagy pathway, PINK1 recruits and activates MUL1, and activated MUL1, in turn, stabilizes PINK1 [114]. The above results illustrate the multiple roles of MUL1 in the autophagy pathway.

#### 2.1.4. MARCH5

##### MARCH5 and Chaperone-Mediated Autophagy (CMA)

Membrane-associated ring-CH-type finger 5 (MARCH5), located in the OMM, is ubiquitously expressed in various human tissues, including the heart, brain, liver, and lung. MARCH5 consists of four predicted transmembrane domains (TMs) and an N-terminal C4HC3-type RING finger domain that is critical for ubiquitin ligase activity [115,116]. MARCH5 is a regulatory factor responsible for mitochondrial quality control, and its defects will impair mitochondrial function and lead to various diseases, including cancers and neurodegenerative disease [117].

As previously mentioned, the E3 ligase Parkin regulates mitochondria through the macroautophagy pathway; however, it is worth noting that chaperone-mediated autophagy (CMA) represents an alternative mechanism by which E3 ligases participate in mitochondrial regulation. CMA is highly selective and often utilizes chaperone protein Hsc70 to specifically degrade target proteins with unique “KFERQ (Lys-Phe-Glu-Arg-Gln)” motifs [118,119]. Multiple studies have shown that the activity of CMA may be impaired in PD and thus contributes to the pathogenesis of PD. The regulation of mitochondria by CMA is achieved through MARCH5.

MARCH5 is a CMA substrate, and enhancing CMA significantly facilitates MARCH5 turnover. To be specific, the oxidative-stress-induced accumulation of MARCH5 promotes the recruitment of DRP1 into mitochondria, triggering excessive fission of mitochondria in dopaminergic (DA) neurons. This results in impaired dopamine secretion and contributes to the pathogenesis and progression of PD. However, enhanced CMA inhibits the aggregation of DRP1 by promoting MARCH5 turnover, and reverses excessive mitochondrial fission under oxidative stress, thus alleviating the pathogenesis and progression of PD [19]. Since several PD-related proteins, including PINK1, LRRK2, and VPS35, induce excessive fission by increasing the mitochondrial translocation of DRP1, promoting the turnover of MARCH5 is vital for inhibiting the progression of PD.

The excessive accumulation of MARCH5 in mitochondria can promote excessive mitochondrial fission, leading to dysfunction of the mitochondrial machinery. However, MARCH5 has also been identified as a protective factor under stress. For instance, under the condition of hypoxia, the mitochondrial outer membrane protein FUN14 domain containing 1 (FUNDC1) is activated. Through the interaction of LC3, FUNDC1 facilitated the recruitment of autophagy membrane vesicles to envelop mitochondria, thereby orchestrating mitophagy [120]. MARCH5 can induce the ubiquitination and degradation of FUNDC1, thereby reducing mitochondrial sensitivity to hypoxia-induced mitophagy [82]. As mentioned above, Parkin contributes to cell survival. Interestingly, Koyano et al. discovered that damaged mitochondria recruited and activated Parkin through a potential mechanism involving MARCH5-mediated ubiquitination followed by PINK1-mediated phosphorylation, thereby facilitating mitophagy. Moreover, the absence of *MARCH5* diminished the recruitment of Parkin to depolarized mitochondria and its catalytic ubiquitylation activity [20]. The depletion of MARCH5 attenuated Parkin recruitment to OMM and Parkin-catalyzed ubiquitylation [20]. These findings highlight the need for revealing more key factors that fine-tune MARCH5 protein levels or activity under various stress conditions.

#### 2.1.5. DUBs and Mitophagy

As part of the UPS, DUBs also play a critical role in mitophagy. Parkin promotes the poly-ubiquitination of substrates by using K6, K11, K27, and K63 as linkages. These modifications are reversible and can be broken down by Ub-specific protease 30 (USP30), USP33, USP15, and USP8 [121]. USP30 is a member of the USPs deubiquitinating enzyme family, which is mainly localized to the outer mitochondrial membrane and is a cysteine protease. Various studies have shown that USP30 is involved in regulating intracellular mitochondrial morphology by reversing PINK1/Parkin-mediated mitophagy [62,122]. In neuronal cell models, knockdown of USP30 resulted in increased PINK1-mediated phosphorylation of Ub and mitophagy. Overall, the inhibition of USP30 can enhance mitophagy, thereby partially inhibiting the progression of PD [123]. Similarly, the deubiquitinating enzymes USP15 [124], USP33 [63], and USP8 [125] have been shown to antagonize Parkin-mediated mitochondrial ubiquitination and mitophagy. When USP15, USP33, and USP8 are silenced, K6-linked Ub chains bind to the OMM and contribute to the successful completion of mitophagy. Taken together, under neurotoxic stress, the PINK1/Parkin pathway prevents PD by regulating mitophagy, while USP30, USP15, USP33, and USP8 promote PD by eliminating the Parkin-mediated ubiquitination of substrates. Thus, the inhibition of USP30, USP15, USP33, and USP8 has the potential to be used in the treatment of PD. Notably, PINK1-mediated phosphorylation of Ub inhibits the activity of these DUBs. Therefore, a deeper understanding of the PINK1–Parkin–DUB pathway is essential for our understanding of mitochondrial homeostasis.

Overall, we have focused on the multiple roles played by the E3s and DUBs in mitophagy. In the PINK1/Parkin pathway, E3 ligase Parkin and MARCH5 jointly regulate mitophagy and maintain mitochondrial homeostasis. PINK1 is responsible for recruiting and activating Parkin, and MARCH5 is required for the initial steps of Parkin recruitment and activation. However, SIAH1 and SIAH3 promote the inactivation and degradation of PINK1. Parkin activates and ubiquitinates mitophagy-related proteins, eventually leading to mitophagy and clearing abnormal mitochondria. Meanwhile, the Parkin-assembled Ub linkage chain can be disassembled by deubiquitinating enzymes USP30, USP33, USP15, and USP8. In addition, MARCH5 directly degrades the mitophagy protein FUNDC1, reducing the sensitivity of mitochondria to hypoxia-induced mitophagy. In general, Parkin and MARCH5 delay the progression of PD by regulating mitophagy in the autophagic lysosomal pathway, while in contrast, the accumulation of MARCH5 also leads to the aggregation of DRP1 into mitochondria in the CMA pathway, leading to abnormal mitochondrial fission in DA neurons. Beyond this, the mitochondrial E3 ubiquitin ligase MUL1 regulates mitophagy together with the PINK1/parkin pathway and restrains Parkin-mediated mitophagy in mature neurons by maintaining ER–mitochondrial contacts. MUL1 may be a downstream target of the PINK1/parkin pathway in mitophagy, which needs to be verified in more experiments in the future.

### 2.2. E3s Regulate α-Syn Mediated Signaling

**Figure 3:**
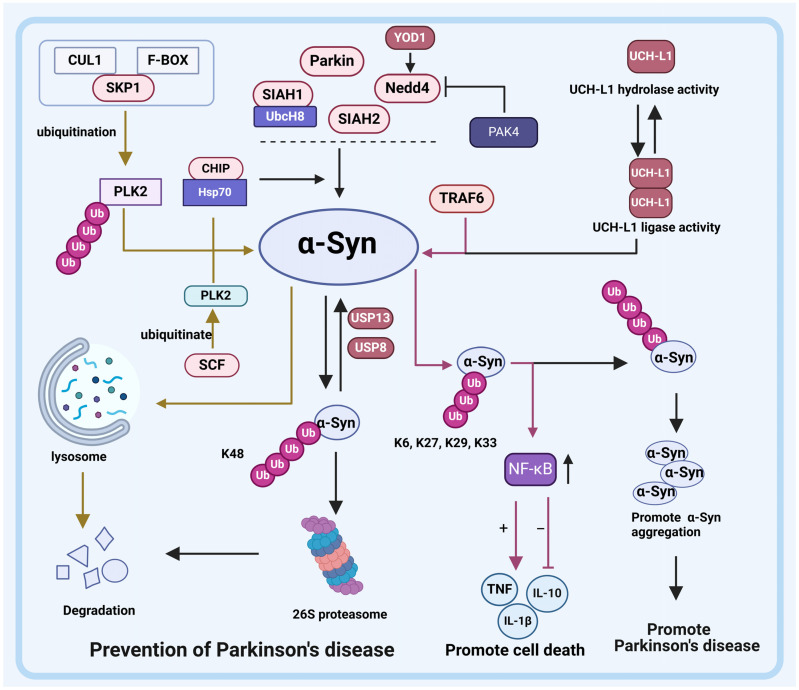
Relationship between E3 ligases and DUBs with α-Synuclein (α-Syn) in PD. E3 ligases SIAH1, SIAH2, Nedd4, CHIP, and Parkin ubiquitinate α-Syn. The degradation of α-Syn by SIAH1 requires the participation of the E2 enzyme UbcH8. USP13 and USP8 can eliminate the ubiquitination of α-Syn mediated by SIAH1, SIAH2, Nedd4, CHIP, and Parkin. Additionally, E3 ligase CHIP also degrades α-Syn through the lysosomal pathway. Similar to CHIP, E3 ligase SCF degrades α-Syn via the lysosomal pathway but requires PLK2’s involvement. The ubiquitination modification of PLK2 by SCF promotes lysosomal degradation of α-Syn. Although E3 ligase TRAF6 does not directly degrade α-Syn itself, it activates NF-κB upon ubiquitinated α-Syn binding and ultimately promotes cell apoptosis. Furthermore, DUBs also regulate α-Syn degradation processes. Specifically, YOD1 enhances Nedd4 activity to promote the degradation of α-Syn. UCH-L1 is a multifunctional DUB that exhibits both deubiquitinase activity and ubiquitin ligase activity in its dimerized state. Dimerized UCH-L1 ubiquitinates α-Syn and facilitates its co-aggregation rather than its degradation; however, the hydrolase activity of UCH-L reverses this process.

As mentioned above, the formation of LBs is one of the primary pathological features of PD. LBs are intracellular inclusions rich in α-Syn. Notably, the abnormal aggregation of α-Syn, the main component of LBs, in neurons of the substantia nigra is critical for the development of PD [126]. α-Syn is a soluble protein predominantly expressed in presynaptic terminals and perinuclear regions of the central nervous system (CNS). It is encoded by the SNCA gene, consisting of 140 amino acids (aa), and has an estimated molecular weight of approximately 19 kDa. α-Syn consists of an N-terminal helical domain, a hydrophobic central region containing the non-Aβ-amyloid component (NAC) sequence, and the C-terminus rich in acidic amino acids. The N-terminal domain mediates α-Syn nucleoprotein binding to the lipid membrane, while the NAC region is thought to be the starting site for α-Syn nucleoprotein self-assembly into amyloid fibrils [127]. The molecular mechanism of α-Syn aggregation in midbrain dopamine neurons is a bottleneck that needs to be broken through in PD research. Notably, a series of studies have found that various E3s participate in the fate of α-Syn, including the seven in absentia homolog (SIAH) family, tumor necrosis factor receptor-associated factors (TRAF6), neural precursor cell expressed developmentally downregulated protein 4 (Nedd4), Parkin, carboxy-terminus of Hsc70 interacting protein (CHIP), and S-phase kinase associated protein 1 (SKP1) [22,23,24,26,27,31,32] (Figure 3).

#### 2.2.1. SIAH Family

As mentioned above, SIAH family proteins regulate mitophagy by degrading PINK1. Moreover, SIAH family proteins are also vital for α-Syn degradation. Esti Liani et al. reported that SIAH family proteins interact with α-Syn, which may partially promote LBs formation. Specifically, the ubiquitination of α-Syn by SIAH1 and SIAH2 promotes aggregation and increases the toxicity of α-Syn but does not promote degradation [22,23,24]. Interestingly, SIAH1 and SIAH2 have also been reported to participate in the mono-ubiquitination of α-Syn; however, it is noteworthy that SIAH1 exerts a more pronounced effect on the ubiquitination of α-Syn compared to SIAH2 [128]. The disparity in ubiquitination of α-Syn is attributed to the differential roles of SIAH1 and SIAH2, with SIAH1 facilitating both mono-ubiquitination and dual-ubiquitination by interacting with UbcH8, while SIAH2 exclusively mediates mono-ubiquitination. Additionally, Lee et al. (2008) investigated the impact of α-Syn mutations on SIAH1-mediated ubiquitination, revealing a decreased binding ability of A30P and A53T mutants to SIAH1 compared to wild type *α-Syn*, and complete abrogation of the ubiquitination effect by the A30P mutant [24].

Notably, SIAH1, in addition to ubiquitinylating α-Syn, has also been shown to interact with synphilin-1 and promote its degradation [25]. However, synphilin-1 interacts with α-Syn to further promote the formation of LBs. In general, the above findings suggest that the inactivation of SIAH may be an important trigger for the formation of LBs and the progression of PD.

#### 2.2.2. TRAF6

Tumor Necrosis Factor Receptor-Associated Factors (TRAF6), a member of the tumor necrosis factor receptor-related factors (TRAFs) family, functions as a RING E3 ligase. Structurally, the TRAF6 protein comprises a RING finger domain and five zinc fingers at its N-terminal region, along with a TRAF domain at its C-terminal region [129,130]. Notably implicated in both genetic and sporadic PD cases, TRAF6 orchestrates the assembly of ubiquitin conjugates consisting of K6-, K27-, K29-, and K33-linked Ub chains onto ubiquitinated substrates [26].

In PD patients, TRAF6 was found to interact with α-Syn to form atypical Ub linkage chains. Zucchelli et al. (2010) experimented on brain samples from two patients, and TRAF6 was detected in almost all substantia nigra LBs in both patients. TRAF6 is detected throughout the cytoplasm of PD patients’ brains but accumulates in the more prominent parts of α-Syn staining in LBs. Atypical Ub linkage chains are not involved in the degradation of proteins. This result is consistent with the fact that α-Syn in LBs is ubiquitinated but not degraded by the 26S proteasome [35]. Subsequently, Yshii. et al. (2020) conducted in-depth research on the role of TRAF6-ubiquitinated α-Syn and found that the interaction between TRAF6 and α-Syn leads to the increased activity of NF-κB. α-Syn itself could activate NF-κB activity, but the ubiquitination of α-Syn by TRAF6 further increases NF-κB activity. Subsequently, the increased activity of NF-κB increases TNF and IL-1β and decreases IL-10, ultimately reducing cell viability and increasing cell death. Interestingly, the interaction of TRAF6 with α-Syn A30P mutant did not have this effect, which may be related to the α-Syn A30P mutant causing its own functional damage. To confirm this, the role of α-Syn A30P mutants in cell models, as well as in vivo, should be further studied [26]. However, to date, no studies have been conducted to investigate the accumulation of α-Syn caused by *TRAF6* mutations in humans. Given the role of TRAF6 in PD, in-depth research on TRAF6 may play an important role in understanding the pathogenesis of PD.

It is also worth noting that SIAH family proteins have domains that are highly similar to the TRAF-C region of the TRAF protein, suggesting that they may have the same regulatory mechanism when ubiquitinating α-Syn [131]. However, this hypothesis needs to be confirmed by further research.

#### 2.2.3. Nedd4

The E3 ubiquitin ligase Neural Precursor Cell Expressed Developmentally Downregulated Protein 4 (Nedd4) is a member of the Nedd4-like family, which belongs to the HECT ubiquitin ligase family. Nedd4 comprises a C2-domain, WW domains, and a HECT-domain at the C-terminus, where the WW domain is commonly used to identify substrates [132,133]. Nedd4 is located in the substantia nigra neurons and is abnormally overexpressed in neurons with LBs. Nedd4 acts as an inhibiting factor in PD via ubiquitination and degradation of α-Syn. The Ub conjugates assembled by Nedd4 consist of K63-linked Ub chains, allowing for the subsequent degradation of ubiquitinated a-Syn by the 26S proteasome. Thus, the overexpression of Nedd4 enhances α-Syn clearance, and the reduction in Nedd4 increases the level of α-Syn, promoting the progression of PD [27]. Additionally, the involvement of p21-activated kinase 4 (PAK4) in the regulation of α-Syn has been confirmed by Won et al., highlighting that PAK4 inactivation leads to α-Syn aggregation [134]. Notably, this process requires the involvement of Nedd4. To be specific, PAK4 acts as an upstream effector of Nedd4 and hinders α-Syn degradation through its interaction with Nedd4, thereby promoting α-Syn aggregation within the brain. Overall, the above results suggest that Nedd4 is a key regulator of α-Syn aggregation, and that induced activation of Nedd4 in neurons may be a potential therapeutic approach to delay the progression of PD.

#### 2.2.4. CHIP

Carboxy-Terminus of Hsp70 Interacting Protein (CHIP), a dual-function cochaperone/E3 ligase protein, is a member of the U-box E3 ligase family. CHIP is mainly localized in the cytoplasm, and consists of a three tetratricopeptide repeat (TPR) domain that binds to Hsc70, Hsp70, and Hsp90, a high-charge central domain, and a U-box domain that has a modified RING-finger motif base with Ub ligase activity [135]. Like SIAH proteins, CHIP is a component of LBs in the human brain, and its overexpression results in reduced levels of a-Syn. That is, CHIP regulates α-Syn levels and aggregation properties by interacting with Hsp70 to direct α-Syn to the UPS degradation pathway [28,136]. Notably, Shin et al. (2005) demonstrated that CHIP facilitates α-Syn transfer to the proteasome pathway through its U-box domain and also mediates α-Syn degradation via the lysosome pathway. Specifically, they observed that both CHIPΔU (U box deletion) and CHIPΔTPR (TRP deletion) exhibited similar reductions in α-Syn levels compared to WT CHIP. Subsequently, they employed the calpain inhibitor ALLN (N-acetyl-Leu-Leu-norleucinal) to inhibit the proteasome pathway. Interestingly, ALLN treatment exclusively blocked CHIPΔU-mediated degradation of α-Syn while full-length CHIP and CHIP-ΔTPR retained their ability to degrade α-Syn, supporting the role of the U-box domain in inducing lysosomal degradation of α-Syn. Furthermore, inhibition of the lysosomal degradation pathway using ammonium chloride NH4Cl revealed that NH4Cl treatment only impaired the activity of CHIPΔTPR in degrading α-Syn but had no effect on WT CHIP or CHIPΔU [29]. These results confirm the involvement of the lysosome pathway in the degradation of α-Syn.

In sum, the presence of CHIP degrades α-Syn through the UPS and lysosomal pathways. Improving the activity of CHIP is expected to contribute to the treatment of PD.

#### 2.2.5. SKP1

S-Phase Kinase Associated Protein 1 (SKP1) is a component of the Skp1/CUL1/F-box (SCF) E3 ubiquitin ligase complex [137], and emerging evidence suggests its involvement in the pathogenesis of PD [138]. To elucidate the role of SKP1 in the regulation of Parkinson’s disease (PD), Zhang et al. (2021) investigated the regulatory mechanism by which SKP1 influences α-Syn proteins. The findings revealed that downregulation of SKP1 impairs α-Syn clearance by attenuating PLK2-mediated ubiquitination. It is well-established that PLK2 interacts with α-Syn and facilitates its autophagic degradation [30]. Furthermore, preclinical studies have demonstrated that point-directed intra-striatal injection of a lentivirus targeting SKP1 can induce pathological and behavioral impairments in mice, providing evidence for the essential role of SKP1 in maintaining viable dopaminergic neurons [139,140].

#### 2.2.6. Parkin

As mentioned earlier, the E3 ubiquitin ligase Parkin plays an essential role in the pathogenesis of PD by regulating mitophagy. Furthermore, Parkin also plays an essential role in the regulation of α-Syn [141]. First, Parkin can prevent α-Syn accumulation by activating protein phosphatase 2A (PP2A). PP2A reduces the formation of toxic oligomers by dephosphorylating pSer262/396/404 tau and pSer129 α-Syn, ultimately reducing LBs and neurofibrillary tangles. Second, Parkin inhibits the activity of PLK2, which is responsible for phosphorylating α-Syn on Ser129 and promoting the formation of α-Syn polymers. In addition, by inhibiting monoamine oxidase B (MAO-B, a key enzyme that degrades dopamine), Parkin leads to a decrease in oxidative stress, which reduces the formation of α-Syn and oligomers [31]. The above pathways work together to inhibit the aggregation of α-Syn and delay the progression of PD. In addition, Parkin promotes the ubiquitination and degradation of glycosylated α-Syn (α-Sp22) by binding to the E2 ubiquitination enzyme UbcH7, but does not act on unmodified α-Syn. Additionally, autosomal recessive PD-associated mutations (Parkin R42P and T240R mutants) lose the ability to bind α-Sp22, resulting in the pathological accumulation of α-Sp22 in the brain [90].

#### 2.2.7. DUBs and α-Syn

As mentioned above, DUBs differ in the regulation of mitophagy. In addition, a variety of DUBs have also been reported to be involved in the degradation of α-Syn, which is closely related to the occurrence and development of PD. Currently reported DUBs associated with α-syn are ubiquitin carboxy-terminal hydrolase L1 (UCH-L1), YOD1, USP13, USP9X, and USP8.

##### UCH-L1

Ubiquitin Carboxy-Terminal Hydrolase L1 (UCH-L1), a DUB of the UCH family, is a protein containing 223 amino acids. UCH-L1 is a multifunctional protein highly explicitly expressed by neurons in the brain. Specifically, UCH-L1 has deubiquitinase activity and exhibits ubiquitin ligase activity when dimerized. The specificity of localization of UCH-L1 enables it to play a role in PD and other neurodegenerative diseases; UCH-L1 has been identified as a risk factor for PD. While UCH-L1 was described initially as a DUB, Liu et al. found that E3 ligase activity displayed by UCH-L1 influences α-Syn degradation and susceptibility to PD [70]. The in vitro study demonstrated that UCH-L1 exhibits ubiquitination activity towards α-Syn and facilitates the co-aggregation of α-Syn rather than its degradation, while the hydrolase function of UCH-L1 counteracts this process. Furthermore, familial mutations and post-translational modifications of UCH-L1 have been observed in the brains of individuals with PD [70]. The UCH-L1 I93M mutant was identified as the cause of autosomal dominant PD. In contrast, the UCH-L1 S18Y mutant was a protective variant. The DUB activity of the UCH-L1 I93M mutant was halved compared to that of WT UCH-L1, while the DUB activity of the UCH-L1 S18Y mutant variant showed an upward regulation trend, which is part of the reason for the different outcomes between the two variants. Subsequent experiments demonstrated that both WT UCH-L1 and the UCH-L1 I93M mutant significantly elevated α-Syn levels (greater than 2-fold), while having a minimal impact on α-Syn levels with the UCH-L1 S18Y mutant. Overall, the pathogenesis of UCH-L1 and UCH-L1 I93M mutants is attributed to a partial loss of UCH-L1 hydrolase activity and an increase in dimerization-dependent E3 ligase activity [70]. In summary, UCH-L1 may exert a regulatory influence on the initiation and progression of PD, potentially offering therapeutic advantages for PD management.

##### YOD1

YOD1 is a DUB from the OTUs subfamily that has been identified to play a role in multiple degenerative diseases such as PD [142]. As mentioned in the previous section, Nedd4 acts as an inhibiting factor in PD via degrading α-Syn. YOD1 was found to promote the degradation of α-Syn and inhibit PD progression by upregulating Nedd4. Thus, the activation of the YOD1-Nedd4-α-Syn signaling axis can especially contribute to inhibiting the pathogenesis of PD [72].

##### USP13/USP9X/USP8

USP13 is identified as a PD promoter by increasing α-Syn levels via two underlying mechanisms. First, USP13 favors the inhibited degradation of α-Syn through autophagy. Second, *USP13* knockdown can increase Parkin ubiquitination and activity, thereby reducing the level of α-Syn; the excessive expression of USP13 increases the expression of α-Syn [68,69]. Similarly, USP8 deubiquitination of the K63 linkage on α-Syn favors the inhibited lysosomal degradation of α-Syn, resulting in the accumulation of α-Syn in LBs [67]. In brief, USP13 and USP8 promote α-Syn-related pathology, and the knockdown of USP13 and USP8 reduces α-Syn-induced DA neuronal death. In addition, USP9X has been reported to be involved in the degradation of α-Syn by reversing SIAH1-mediated mono-ubiquitination [65,66]. In summary, SIAH2, Nedd4, CHIP, and Parkin directly ubiquitinate α-Syn, while SIAH1 mediates the degradation of α-Syn through the mono-ubiquitination and polyubiquitination modifications that necessitate the involvement of E2 enzyme UbcH8. In comparison to SIAH2, Nedd4, CHIP, and Parkin-induced degradation of α-Syn, dual ubiquitination also enhances the effectiveness of SIAH1 in degrading α-Syn. In addition to α-Syn degradation by direct ubiquitination, CHIP can also degrade α-Syn by the lysosomal pathway [29]. Like CHIP, SCF degrades α-Syn via the lysosomal pathway, but this requires the participation of PLK2. The ubiquitination modification of PLK2 by SCF promotes the lysosomal degradation of α-Syn. Unlike the above E3, TRAF6’s ubiquitination of α-Syn does not directly degrade α-Syn; the α-Syn combined with the K6, K27, and K29-linked ubiquitin chains can activate NF-κB and ultimately promote cell apoptosis [35].

Different types of E3 ligases modify α-Syn in different ways, leading to different outcomes. It is still necessary to analyze the relative contribution of different types of E3 to the ubiquitination of α-Syn through further experiments, and determine whether different interventions for different types of E3 ligases can achieve the effect of delaying the progression of PD.

### 2.3. Additional E3-Regulated Signaling Pathways Associated with PD

E3s have also been shown to play a role in PD progression via ubiquitinating elements of some critical cellular pathways, including DJ-1 and Parkin.

DJ-1 is encoded by the PARK7 gene and belongs to the peptidase C56 family. DJ-1 is recognized as a PD-related protein that protects neurons from oxidative stress and cell death, and the deletion or mutation of DJ-1 is one of the causes of autosomal recessive early-onset PD [143]. Current studies have shown that the E3 ubiquitin ligase Parkin and TRAF6 regulate DJ-1. Parkin ubiquitinates pathogenic monomeric forms of DJ-1 (e.g., L166P DJ-1), but interacts with WT DJ-1 only under conditions of oxidative stress. The DJ-1 L166P mutant makes the protein unstable and affects the binding of the DJ-1 protein to its ligand, thereby inducing the occurrence of PD [144]. Parkin facilitates the stabilization of both WT DJ-1 and the DJ-1 L166P mutant at the outer mitochondrial membrane (OMM), leading to elevated cellular levels of DJ-1 through the prevention of degradation, thereby mitigating oxidative stress and promoting a healthy mitochondrial environment [33,34].

Furthermore, the process of TRAF6 ubiquitinating the oxidative stress sensor DJ-1 is also actively involved in the pathogenesis of PD. TRAF6 stimulates the accumulation of DJ-1 L166P mutants into insoluble cytoplasmic aggregates by promoting atypical ubiquitination (K1, K6, K27, and K29-linked ubiquitin chains) of DJ-1 L166P mutants instead of WT DJ-1 to avoid their heterozygous toxicity [35].

As mentioned above, Parkin is a ligase that plays an important role in mitophagy, acting by ubiquitinating multiple substrates. Parkin has also been reported to be regulated by the E3 ligase, CHIP. CHIP can interact directly with Parkin and increase the activity of Parkin [21]. CHIP interacts with Parkin to jointly regulate the pathogenesis of PD.

In addition to promoting the degradation of α-Syn, Nedd4 has been reported to delay PD progression by modulating RTP801. RTP801 is a pro-apoptotic protein that is indispensable in inducing neuronal death in PD. The degradation of RTP801 through direct ubiquitination by Nedd4 reduces neuronal toxicity [36].

## 3. Targeting E3 Ligases and Deubiquitinating Enzymes for PD Therapy

At present, the main treatment for PD includes four types, as follows: sports rehabilitation, medication, psychotherapy, and surgery. The traditional treatment for PD is a pharmacological dopamine substitution strategy involving carbidopa/levodopa or dopamine agonists [145]. Considering that the pathogenesis of PD primarily involves abnormalities in the ubiquitin–proteasome system and the dysregulation of mitochondrial function, modulation of E3 ligases and DUB activity holds therapeutic potential for PD treatment. Currently, research on E3 ligase and DUB inhibitors for PD predominantly focuses on putative targets and primarily conducts fundamental investigations, with a dearth of clinical trials and applications pertaining to E3 ligase and DUB inhibitors. (Table 4).

Notably, the use of proteolytic-targeted chimeras (PROTACs) is a novel drug design strategy for the treatment of diseases. PROTACs are bifunctional hybrid molecules composed of a target protein-binding ligand, an E3 ubiquitin ligand and a linked chain. The mechanism of action of PROTACs is to achieve the effect of drugs by combining protein degradation mechanisms with the binding of specific proteins [146]. PROTACs can bind to the target protein at one end and E3 at the other, thereby recruiting the target protein to E3. In this way, PROTACs can reduce the levels of abnormal proteins, thereby reducing their toxic effects or preventing their participation in disease processes. One of the biggest advantages of PROTACs is that they can be recycled, allowing for proteins to be targeted more efficiently and thereby allowing for highly selective treatments [146]. In contrast to conventional drugs, PROTACs can inhibit their function by degrading their target proteins, rather than by inhibiting their activity alone. This unique mechanism may lead to better treatment outcomes and lower side effects. PD has the potential to benefit from PROTACs in the future. PROTACs can be applied to degrade aberrantly aggregated α-Syn. By designing a highly selective PROTACs molecule, it can bind to α-Syn and direct it to the cell’s protein degradation system, thereby promoting the degradation of α-synuclein. By degrading aberrantly aggregated α-Syn, PROTACs have the potential to reduce the occurrence of toxic effects in Parkinson’s disease and slow disease progression. This strategy can fundamentally intervene in the pathological mechanism of PD and may provide new options for the treatment of Parkinson’s disease. Although further research and clinical trials are still needed in practical applications, PROTACs have great potential as a potential drug design strategy for the treatment of PD. At present, the PROTAC strategy has been applied to fight some neuro-degenerative diseases. For example, a groundbreaking study of the effects of PROTACs on Alzheimer’s disease was successful in 2016 and demonstrated that TH006 abolishes the activity of tau by recruiting von Hippel–Lindau (VHL) E3 ligase, Subsequently, different groups developed Tau-Keap1-CPP, QC-01-175, and C004019 PROTACs using Keap1, CRBN, and VHL E3, respectively, as ligases to target the degradation of tau in Alzheimer’s disease models. In addition, R.B. Kargbo has developed PROTACs for tau and α-Syn that contain CRBN and VHL E3 ligases, which may be used in the treatment of PD [147]. It is important to note that PROTACs are a relatively new drug design strategy and are still in the research and development phase. While it has great potential in terms of potential therapeutic applications, more research and clinical trials are needed to validate its safety and efficacy.

**Table 4:** Inhibitors of E3 ubiquitin ligases and deubiquitinating ligases in PD.

Targets	Compound	Mechanism of Action	Global Status	Molecular Structure	Reference
Nedd4	Ubv.N4.02	Improve the ubiquitination of Nedd4 and thus degrade substrates	Preclinical	Inapplicability	[148,149]
SKP1	MLN4924	Inhibits E2 and CUL1, blocks SCF complex assembly	Preclinical	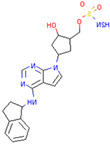	[150,151]
SIAH1/2	vIRD	Binds to SKP1-CUL1 complexes and thus affects the activity of SCF	Preclinical	Inapplicability	[152]
	HCF1, HCF2	Substrate binding domain (SBD) that binds and blocks SIAH1/2	Preclinical	Inapplicability	[153]
	Adapalene	Inhibits SIAH2 ubiquitin ligase activity and alters HIF1α activity	Preclinical	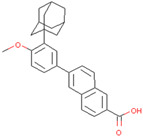	[154]
TRAF6	Bortezomib	Inhibition of function and maturation of multiple myeloma osteoclast by downregulating TRAF6	Preclinical	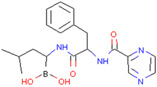	[155]
	MG132	Treat pancreatic cancer by inducing autophagy and downregulating TRAF6 in combination with radiation	Preclinical	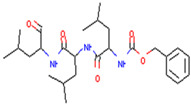	[156]
	C25-140	Reduces the activity of TRAF6-Ubc13, thereby inhibiting the production of ubiquitin chains linked to Lys63	Preclinical	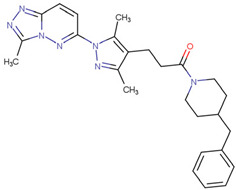	[157]
	EGCG	Binding with TRAF6 inhibits its activity	Preclinical	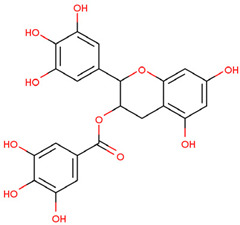	[158]
	Resveratrol	Mediates the degradation of TRAF6 and inhibits the action of EMT	Preclinical	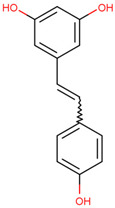	[159]
	TRAF6 inhibitory peptides	Binds to the T6DP motif of RANK to target TRAF6	Preclinical	Inapplicability	[160]
	TRAF-2019 inhibitor peptide	Reduces Tregs in tumors and prevents Tregs from migrating into tumors	Preclinical	Inapplicability	[160]
	Shikonin	Prevents interaction between TRAF6 and RANK	Preclinical	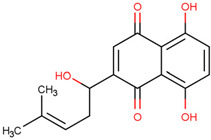	[161]
	Nodakenin	Disturbance of activation of TRAF6 in macrophages to block activation of NF-κb	Preclinical	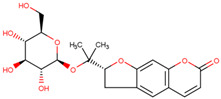	[162]
USP30	MF-094	Suppressed NLRP3 inflammatory bodies	Preclinical	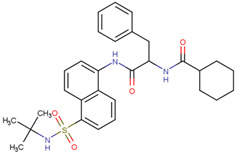	[163]
	FT385	Adducts formed with USP30 induce mitochondrial autophagy in SH-SY5Y cells	Preclinical	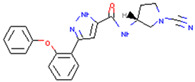	[164]
	Imidazole phenoxyacetic acids	Inhibits apoptosis of SH-SY5Y cells	Preclinical	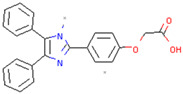	[165]
	MTX652	Promotes damaged mitochondrial degradation to treat chronic kidney disease	Phase II clinical trial	Unreported	[166,167]
	15-oxospiramilactone	Effectively induces mitochondrial fusion and restores oxidative respiration and mitochondrial networks in cells lacking Mfn1 or Mfn2	Preclinical	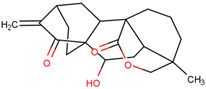	[168]
UCH-L1	LDN91946	Binds to the Michaelis complex, inhibits the multiplication of stromal tumor cells	Preclinical	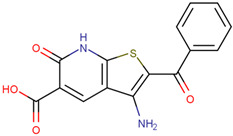	[169,170]
	LDN57444	Improves leiomyoma cell migration, gel contraction and collagen synthesis, and reverses UCH-L1 overexpression of cardiac hyperplasia	Preclinical	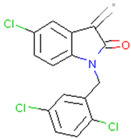	[171,172]
USP13	Spautin-1	Promotes cell death and inhibits autophagic cell death under starvation conditions	Preclinical	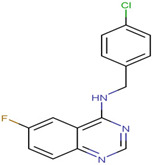	[173]
USP9X	WP1130	Inhibits USP9X activity	Preclinical	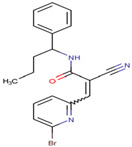	[174]
	EOAI3402143	Inhibits USP9X activity	Preclinical	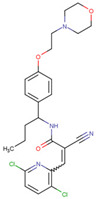	[175]
USP8	RA-9	Reduces HeLa cell viability	Preclinical	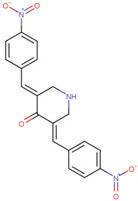	[176]
	RA-14	Induces apoptosis of ES-2 cells	Preclinical	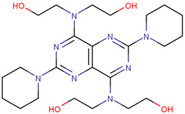	[177]
	AM146	Reduces cell viability	Phase II clinical trial	Unreported	[176]
USP14	IU1	Promotes the degradation of tau and ataxia-3 proteins, reduces menaquinone-induced oxidative protein accumulation, improves menaquinone-induced human HEK293 cell death, and corrects damaged mitochondrial autophagy targeting PD	Preclinical	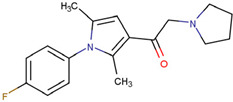	[61,178]
	B-AP15	Inhibits the growth of WM and MM cells and induces their apoptosis, overcoming bortezomib resistance	Preclinical	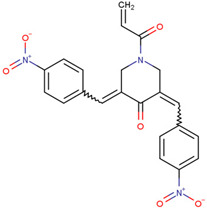	[179,180,181]
	VLX1570	Inhibits the growth of MM cells and induces their apoptosis	Discontinued	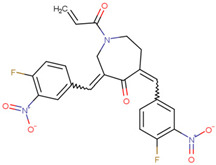	[179,180,181]

### 3.1. Targeting E3 Ligases for PD Therapy

#### 3.1.1. Nedd4

As mentioned above, Nedd4 can further inhibit the development of PD by ubiquitinating α-Syn and eliminating toxic proteins. Therefore, increasing the activity of Nedd4 may be a therapeutic strategy in PD therapy; a Nedd4 activator named Ubv.N4.02 was found to improve ubiquitination by Nedd4 of its substrates [148,149]. However, there are currently no studies on applying Nedd4 activators in PD. Moreover, the development of related PROTAC and recruiting target proteins to Nedd4 to promote their degradation may become a new treatment strategy.

#### 3.1.2. SKP1

As discussed, SKP1 is a component of the SCF E3 ubiquitin ligase complex, which inhibits the clearance of α-Syn by weakening the ubiquitination of PLK2. Currently, drugs targeting SCF include MLN4924 and vIRD. MLN4924 selectively inhibits SCF functions, including the inhibition of E2 binding and function; blocking SCF complex assembly, and blocking the interaction between substrate and F-box proteins by inhibiting CUL1 [150]. MLN4924 is undergoing Phase I clinical trials to treat several human malignancies, including melanoma [151]. In addition, the viral inhibitor vIRD has been identified, which can bind to the SKP1-CUL1 complex during vaccinia virus infection and thus affect the activity of SCF [152]. However, the exact mechanisms behind them have yet to be studied. Unfortunately, no studies have been conducted on SCF inhibitors in PD, but whether MLN4924 can inhibit SCF in PD is worth exploring.

#### 3.1.3. SIAH1/2

As mentioned above, SIAH1 and SIAH2 play an essential role in the development of PD. SIAH1 and SIAH2 promote ubiquitination of α-Syn, which promotes α-Syn aggregation in vitro and vivo, leading to increased toxicity.

To date, the known inhibitors of SIAH1/2 are at the primary research stage and have not been put into clinical trials and applications. HCF1 and HCF2 can inhibit the activity of SIAH1/2 and bind and block the substrate binding domain (SBD) of SIAH1/2 to prevent deubiquitination and self-ubiquitination of downstream targets [153]. Small-molecule SIAH1/2 inhibitors have been screened such as RLS-7, adapalene and menaquinone. In melanoma and prostate cancer cells, adapalene potently inhibits SIAH2 ubiquitin ligase activity in vitro and alters HIF1α activity [154]. Notably, the presence of SIAH1/2 in LBs suggests that SIAH1/2 may become a new therapeutic target in PD treatment strategies. Existing SIAH1/2 inhibitors are being trialed as cancer therapies but have not been researched in PD.

#### 3.1.4. TRAF6

As described, researchers have found that TRAF6 and α-Syn coexist with LBs in the postmortem brain tissue of PD patients [182]. TRAF6 has a closed link to α-Syn and DJ-1, which promotes the entry of DJ-1 mutant with polyubiquitination into the cytoplasm [35].

Inhibitors targeting TRAF6 have been widely studied; these can be divided into protease inhibitors, TRAF6-inhibiting peptides, small-molecule inhibitors targeting TRAF6, and herbal extracts. Bortezomib and MG132 are protease inhibitors that inhibit tumor development by downregulating the expression of TRAF6. Both are at the primary research stage and have not yet been put into clinical trials. Bortezomib is a potential drug for treating myeloma bone disease by downregulating TRAF6 to inhibit the function and maturation of osteoclasts in multiple myeloma, and MG132 can be used in combination with radiotherapy to treat pancreatic cancer by inducing autophagy and downregulating TRAF6 [155,156]. Because the proteasome is critical in degrading protein aggregates in PD, this proteasome inhibitor is currently only being studied in tumors and is not the drug of choice for the treatment of PD

C25-140, epigallocatechin-3-gallate (EGCG), and resveratrol are small-molecule inhibitors targeting TRAF6. Compound C25-140 reduces the activity of TRAF6-Ubc13, thereby inhibiting the production of Ub chains linked to Lys63 [157]. EGCG is used in the prevention and treatment of melanoma by targeting TRAF6 [158]. Resveratrol may mediate the degradation of TRAF6 and inhibit the action of epithelial–mesenchymal transition (EMT), thereby reducing the proliferation and migration of prostate cancer cells [159].

TRAF6-mediated signal transduction is inhibited by peptide disruption of the TRAF6 receptor, which is specific for the TRAF6-binding domain. In addition, TRAF6 bait peptide (T6DP)-inhibited RANKL-mediated osteoclast production and bone resorption [183]. The TRAF-2019 inhibitor peptide inhibited tumors in immunocompetent C6BL/57 mice by reducing Tregs in tumors and preventing Tregs from migrating to tumors [160].

Shikonin and Nodakenin are both herbal extracts. Shikonin prevents the interaction of TRAF6 and RANK [161]. Nodakenin prevents NF-κB activation by interfering with the activation of TRAF6 in macrophages [162]. However, neither has been studied in PD. Although many TRAF6 inhibitors have been studied to date, they have not yet been established in clinical trials, and their application in PD has not been found.

### 3.2. Targeting DUBs for PD Therapy

In recent years, effective and specific small-molecule inhibitors of DUBs have been developed, and it has been established that some DUB inhibitors are available in neurodegenerative diseases.

#### 3.2.1. USP30

USP30 antagonizes PINK1/Parkin-mediated mitochondrial autophagy [62,122]. At present, many compounds form covalent or noncovalent bonds with cysteine at the USP30 active site to inhibit USP30 activity and increase mitochondrial autophagy such as WO2019071073A1, WO2020072964A1, O2018213150A1. Moreover, inhibitors of USP30 also include MF-094, FT385, imidazole phenoxyacetic acids, MTX652and 15-oxospiramilactone. MF-094 accelerates the wound healing of diabetes by inhibiting the NLRP3 inflammasome [163]. FT385 contains a cyanoamide group that increases the ubiquitination of TOM20 and induces mitochondrial autophagy in SH-SY5Y cells by forming adducts with USP30 [164], while imidazole phenoxyacetic acids inhibit the apoptosis of homologous cells [165]. Excitingly, MTX652, which promotes the degradation of damaged mitochondria for the treatment of chronic kidney disease, has entered a Phase II clinical trial [166,167]. 15-oxospiramilactone affects the occurrence and development of neuropathy by effectively inducing mitochondrial fusion and restoring oxidative respiration and the mitochondrial network in cells lacking Mfn1 or Mfn2 [168]. Since the deficiency of Mfn2 ubiquitination is associated with PD, in-depth studies of 15-oxospiramilactone will provide a promising pathway for PD therapeutic development.

#### 3.2.2. UCH-L1

As stated before, UCH-L1 affects sensitivity to PD by ubiquitinating α-Syn and promoting α-Syn coaggregation [70]. Inhibitors of UCH-L1 include LDN91496 and LDN57444. LDN91946 is a non-competitive UCH-L1 inhibitor with moderate potency that does not bind to free enzymes and only binds to the Michaelis complex (enzyme-substrate complex); it attenuates the proliferation of mesothelioma and lung cancer cell lines [170]. In addition, LDN57444 is a reversible active UCH-L1 site-specific inhibitor that improves leiomyoma cell migration, gel contraction, and collagen synthesis, and reverses UCH-L1 overexpression of cardiac hyperplasia [171,172]. However, there have been no studies on SCF inhibitors in PD, but given the effect of SCF inhibitors in cancer, it may be possible to use SCF inhibitors in PD in the future.

#### 3.2.3. USP13/USP9X/USP8

As mentioned above, USP13, USP9X, and USP8 all affect the degradation of α-Syn. Current inhibitors for USP13, USP9X, and USP8 include Spautin-1, WP1130, EOAI3402143, RA-9, RA-14, and AM146. Compound Spautin-1 targets the USP13, which promotes cell death and inhibits autophagic cell death under starvation conditions [173]. WP1130 and EOAI3402143 are inhibitors of USP9X, inhibiting its activity and inducing apoptosis in tumor cells [174,175]. Inhibitors of USP8 include RA-9, RA-14, and AM146. RA-9 and AM146 reduced cell viability [176], while RA-14 induced apoptosis of ES-2 cells, improved the overall survival rate of mouse carrying ES-2 tumors, and inhibited ovarian cancer growth [177]. However, none of these inhibitors have been studied in PD. In the context of tumors, all these inhibitors led to cell death and apoptosis. However, since most cells die excessively in neurodegenerative diseases, it is important to explore the effects of these inhibitors on nerve cells.

#### 3.2.4. USP14

USP14 inhibits mitophagy and regulates proteasome activity through deubiquitinating proteasome-binding substrates, thereby influencing the progression of PD [184]. Moreover, USP14 inhibitors include IU1, B-AP15 and its analogue, VLX1570. IU1 promotes the degradation of the neurodegenerative disease-associated proteins, Tau and ataxin-3, reduces menaquinone-induced oxidative protein accumulation, and improves menaquinone-induced human HEK293 cell death [184]. Notably, in the established Parkin and PINK1 mutated fruit fly models, IU1 corrected mitochondrial dyskinesia and dysfunction. Multiple studies proved that IU1 was not toxic to neuronal populations, indicating that IU1 can treat PD associated with impaired mitophagy [61,178]. Moreover, both B-AP15 and its analogue VLX1570 inhibit the growth of cancer cells and induce apoptosis of cancer cells, while B-AP15 can also overcome bortezomib resistance; studies have shown anticancer effects against solid tumors and multiple myeloma in vivo [179,180,181]. However, VLX1570 failed in clinical trials against multiple myeloma due to patient death, owing to severe dose-associated toxicity.

In summary, the UPS is widely involved in various physiological processes of cells, such as transcription, apoptosis, and autophagy, and their dysfunction is closely related to PD. E3 and DUB inhibitors have the advantages of precision and efficiency, and some inhibitors have been put into clinical trials and shown good efficacy. Although there are currently no drugs explicitly targeting PD, the functions of IU1 and 15-oxospiramilactone are related to the pathogenesis of PD, and are worth exploring in the future. In addition, PROTAC technology provides new ideas for the development of E3 ubiquitin ligase-targeted drugs. Therefore, understanding the molecular basis and mechanism of PD for therapeutic breakthroughs is of great significance.

## 4. Conclusions and Perspectives

PD is a prevalent neurodegenerative disorder with an intricate pathogenesis. The processes of mitophagy and abnormal aggregation of α-Syn in LBs play crucial roles in the pathophysiology of PD. This review article discusses the E3 ligases and DUBs that regulate these two key factors, and offers insights into potential novel treatments for PD.

The E3 ligases involved in mitophagy, such as Parkin, SIAH1, SIAH3, MARCH5, and MUL1, play crucial roles in maintaining the stability of neuronal cells. Mutations or deletions of Parkin can lead to abnormal aggregation of PD-related proteins, α-Syn and synphilin-1, resulting in cell dysfunction and neuronal death [90,185,186]. The Parkin-mediated mitophagy pathway has significant implications for PD. In the MARCH5/PINK1/Parkin mitophagy pathway, MARCH5 and PINK1 facilitate the recruitment of Parkin into the outer mitochondrial membrane (OMM), where atypical ubiquitination of OMM proteins enables the recognition and activation of damaged mitochondria within the autophagy system [19]. Conversely, members of the SIAH E3 ligase family have an inhibitory effect on Parkin-mediated mitophagy. Ubiquitination by SIAH1 reduces parkin activation and blocks the mitophagy pathway; meanwhile, co-localization with PINK1 leads to inactivation of PINK1 [16]. Moreover, it has been reported that the E3 ligase MUL1 suppresses Parkin-mediated mitophagy by maintaining ER contact with mitochondria [17]. Notably, PINK1 has been demonstrated to recruit and activate MUL1 for the ubiquitination of mitochondrial proteins and the induction of ubiquitin-mediated mitophagy [114]. Thus, MUL1 not only inhibits Parkin-mediated mitophagy but can also be recruited by PINK1 to orchestrate mitophagy. MUL1 may exhibit a dual role in the pathogenesis of PD, necessitating further comprehensive investigation to ascertain its predominant function.

The primary constituents of LBs, α-Syn, are regulated by a diverse array of E3 ligases, including Parkin, SIAH1, SIAH2, SKP1, Nedd4, CHIP and TRAF6. It is well-established that the presence of Parkin can counteract the effects of an abnormal accumulation of α-Syn. Conversely, recent research has indicated that α-Syn oligomers impact Parkin expression, post-translational modification, and activity. Consequently, disruption of the dynamic equilibrium between the Parkin and α-Syn pathways may precipitate PD. In addition to Parkin, the remaining regulatory E3 ligases for α-Syn can be broadly categorized into two groups, as follows: those that promote α-Syn accumulation (including SIAH1, SIAH2, and SKP1) and those that facilitate its turnover (such as Nedd4 and CHIP) [22,23,24,28,136]. Furthermore, the regulation of α-Syn by TRAF6 also enhances NF-κB activity and promotes apoptosis. Additionally, various DUBs play a role in PD pathogenesis, primarily through their action on removing Ub linkage chains assembled by E3 ligases, resulting in a dynamic regulation with E3 ligases.

In conclusion, various E3s participate in the regulation of PD and play different functional roles in mitophagy and α-Syn degradation. Combined with the above, Parkin, the SIAH family, and CHIP work together to regulate mitophagy and the dynamic balance of α-Syn. Notably, some E3s have been reported to be mutually regulated. For instance, CHIP is involved in the regulation of Parkin by interacting with and enhancing its regulatory activity. The interaction between Parkin and CHIP links the two crucial processes of mitophagy and α-Syn degradation, both of which act as genuine inhibitors of PD.

Furthermore, certain metallic elements have been implicated in the risk of developing PD, with one mechanism involving the disruption of metal homeostasis and its impact on cellular protein degradation pathways. Metal homeostatic disruption refers to the imbalance of metal ions (such as copper, iron, and manganese) within the body, leading to the abnormal accumulation of these ions in cells and resulting in excessive production of free radicals and oxidative stress substances that cause damage to cell structures and functions [187]. The association between metal homeostatic disruption and PD encompasses various aspects. One area of research focuses on the aggregation of α-Syn. While copper and iron are essential trace elements for human health, high concentrations may exert harmful effects on the brain [188]. Studies indicate that metal ions (e.g., copper and iron) can interact with α-synuclein, promoting the formation of abnormal aggregates that lead to neuronal damage and cell death, thereby contributing to the pathogenesis of PD [188,189,190]. The dysregulation of metal homeostasis disrupts the intracellular balance of metal ions, leading to their excessive accumulation and the induction of oxidative stress. Consequently, this can upregulate the generation of free radicals, resulting in further impairment of cellular structure and function. Moreover, dyshomeostasis disrupts the intracellular metal ion balance, resulting in excessive accumulation and oxidative stress. This imbalance, and oxidative stress, can lead to heightened free radical production, further compromising cellular structure and function [191]. Simultaneously, dysregulation of metal homeostasis has also been documented to be linked with inflammatory response and immune system activity. Certain metal ions have the ability to stimulate immune cells and inflammatory responses, resulting in increased cytokine release and further provoking an inflammatory reaction in the nervous system, exacerbating the progression of Parkinson’s disease [192].

It is worth mentioning that the dysregulation of metal homeostasis can impact the protein degradation pathways within cells and is closely associated with the risk of PD. It is widely recognized that cells eliminate abnormal proteins through two primary protein degradation pathways, namely the UPS and autophagy. Research has shown that Ub has the ability to bind Cu (II) and Zn (II), thereby impeding the interaction between Ub and Ub-coupled E2 enzymes [193]. At the same time, Cu (II) ions inhibited the three peptidase activities of the isolated 20S proteasome and showed reduced proteasome activity in HeLa cells [194]. Furthermore, iron ions have also been implicated in the pathogenesis of UPS damage in PD. The iron regulatory protein (IRP)/iron response element system contributes to UPS injury-mediated DA neuronal damage, and both iron chelating agents and genetic iron chelating agents have demonstrated neuroprotective effects against proteasome inhibitor-induced DA neuronal degeneration [195,196]. It is crucial to acknowledge the intricate relationship between metal homeostasis and PD, as there are numerous other contributing factors and mechanisms at play. Further investigation is imperative to delve deeper into and elucidate the specific details and mechanisms of this association. This area may hold great promise for future research on Parkinson’s pathogenesis.

In sum, the mechanism by which E3s are involved in PD is quite complex, and how E3s are balanced in the different pathways is unclear. Future research should focus on the priority and extent of various roles of E3 in the process of mitophagy, which contributes to the formation of a complete mitochondrial E3 regulatory network.

To date, the UPS has garnered increasing attention as a potential target for the treatment of PD. Various E3 ligases and DUB inhibitors have been investigated for their therapeutic potential in PD. Regrettably, compounds targeting PD-associated E3 ligases and DUBs are predominantly in the preclinical stage, highlighting the need to develop practical and clinically viable modulators as prospective efficacious treatments for PD.

## Figures and Tables

**Table 1:** Summary of E3 ubiquitin ligases involved in Parkinson’s disease.

Type	E3 Ubiquitin Ligase	Localization	Effect	Reference
Autophagy mediated pathway				
RBR E3 ligase	Parkin	Cytoplasm, Mitochondrion	Parkin activates OMM-related proteins that promote autophagy	[15]
RING E3 ligase	SIAH family	Cytoplasm, Nucleus	SIAH1 targeting the ubiquitination of PINK1. SIAH3 collocates with PINK1 and initiates the inactivation of PINK1.	[16]
RING E3 ligase	MUL1	Mitochondrion, Neuronal cell body, Peroxisome	MUL1 restrains Parkin-mediated mitophagy in mature neurons by maintaining ER-mitochondrial contacts. MUL1 stabilizes PINK1.	[17,18]
RING E3 ligase	MARCH5	Mitochondrion outer membrane, Endoplasmic reticulum membrane	MARCH5 promotes the recruitment of DRP1 into mitochondria, triggering excessive fission of mitochondria. MARCH5 promotes Parkin recruitment in mitophagy. MARCH5 reduce mitochondrial sensitivity to hypoxia-induced mitophagy.	[19,20]
U-box E3 ligase	CHIP	Cytoplasm, Endoplamic reticulum, Mitochondrion	CHIP enhances p activity	[21]
α-Syn mediated pathway				
RING E3 ligase	SIAH family	Cytoplasm, Nucleus	SIAH1 and SIAH2 promote aggregation of α-Syn. SIAH1 promote synphilin-1 degradation.	[22,23,24,25]
RING E3 ligase	TRAF6	Cytosol, Nucleus	TRAF6 ubiquitinated α-Syn and enhance NF-κB activity.	[26]
HECT E3 ligase	Nedd4	Cytoplasm, Plasma membrane, Subsynaptic reticulum	Nedd4 promotes the degradation of α-Syn.	[27]
U-box E3 ligase	CHIP	Cytoplasm, Endoplasmic reticulum, Mitochondrion	CHIP promotes α-Syn degradation through UPS and lysosome pathways	[28,29]
RING E3 ligase	SKP1	Cytosol, Nucleus	SKP1 inhibited the clearance of α-Syn by weakening the ubiquitination of PLK2	[30]
RBR E3 ligase	Parkin	Cytoplasm, Mitochondrion	Parkin inhibit the aggregation of α-Syn. Parkin promotes ubiquitination and degradtion glycosylated form of α-Syn (α-Sp22)	[31,32].
DJ-1 mediated pathway				
RBR E3 ligase	Parkin	Cytoplasm, Mitochondrion	Parkin ubiquitinates DJ-1 and increases homeostatic levels of DJ-1.	[33,34]
RING E3 ligase	TRAF6	Cytosol, Nucleus	TRAF6 stimulates the accumulation of L166PDJ-1 mutants into insoluble cytoplasmic aggregates and to avoid their heterozygous toxicity.	[35]
Other E3 ubiquitin ligases				
U-box E3 ligase	CHIP	Cytoplasm, Endoplasmic reticulum, Mitochondrion	CHIP interact with Parkin and increase the activity of Parkin.	[21]
	Nedd4	Cytoplasm, Plasma membrane, Subsynaptic reticulum	Nedd4 reduces neuronal toxicity downregulate RTP801.	[36]

**Table 2:** Summary of deubiquitinating ligases involved in Parkinson’s disease.

Type	Deubiquitinating Ligases	Effect	Reference
Autophagy mediated pathway			
USP	USP8	USP8 deubiquitinates parkin, thereby regulating mitochondrial autophagy	[60]
USP	USP14	USP14 inhibits mitochondrial autophagy via Drp1 and Mfn2	[61]
USP	USP30	USP30 deubiquitinates parkin, thereby inhibiting mitochondrial autophagy	[62]
USP	USP33(VDU1)	USP33 deubiquitinates Parkin, thereby inhibiting mitochondrial autophagy	[63]
MJDs	ataxin-3	Ataxin-3 inhibites autophagy degradation of Parkin	[64]
α-Syn mediated pathway			
USP	USP9X	USP9X inhibits autophagic degradation of α-Syn	[65,66]
USP	USP8	USP8 deubiquitinates α-Syn, reducing clearance	[67]
USP	USP13	USP13 deubiquitinates α-Syn.	[68,69]
UCH	UCH-L1	UCH-L1 ubiquitinates α-Syn and promotes its coaggregation	[70]
OTU	OTUB1	OTUB1 is colocated with α-Syn	[71]
OTU	YOD1	YOD1 promotes the degradation of α-Syn and upregulating Nedd4.	[72]
Other Deubiquitinating ligases			
USP OTU	USP14 OTUD3	USP14 regulates proteasome activity by deubiquitinating proteasome-binding substrates OTUD3 stabilizes the IRP2 protein to regulate iron homeostasis in the brain	[61] [73]

**Table 3:** Summary of substrates of Parkin.

Targets for Ubiquitylation	Property	Reference
CDCrel-1(Cdcre-1 is a synaptic vesicle-associated protein)	Synaptic vesicle-associated septins protein	[89]
CDCrel-2	Synaptic vesicle-associated septins protein	[89]
α-synuclein(α-Syn)	The main components of LBs	[90]
synphilin-1	The α-Syn interacting protein	[89]
the putative G protein-coupled Parkin-associated endothelin-like receptor (Pael-R)	A protein leading to cytotoxicity and dopaminergic neuronal apoptosis	[31]
α-tubulin	the microtubule protein	[91]
β-tubulin	the microtubule protein	[91]
cyclin E	the cell-cycle protein	[92]
Dopamine transporter (DAT)	the dopamine transporter	[93]
voltage dependent anion channel 1 (VDAC1)	The outer mitochondrial membrane protein	[15]
mitofusion (Mfn)	The outer mitochondrial membrane protein	[15]
Mitochondrial Rho (Miro)	The outer mitochondrial membrane protein	[94]
Hexokinase I (HK 1)	The outer mitochondrial membrane protein	[15]
CDGSH iron sulfur domain 1 (CISD1)	The outer mitochondrial membrane protein	[95]
translocase of outer mitochondrial membrane 20 (TOMM20)	The outer mitochondrial membrane protein	[15]

## Data Availability

Not applicable.
